# Patient Recovery from COVID-19 Infections: Follow-Up of Hair, Nail, and Cutaneous Manifestations

**DOI:** 10.1155/2021/5595016

**Published:** 2021-06-19

**Authors:** Rattapon Thuangtong, Nasikarn Angkasekwinai, Charussri Leeyaphan, Daranporn Triwongwaranat, Kanchalit Thanomkitti, Kanyalak Munprom, Kanokvalai Kulthanan

**Affiliations:** ^1^Department of Dermatology, Faculty of Medicine Siriraj Hospital, Mahidol University, Bangkok, Thailand; ^2^Division of Infectious Diseases and Tropical Medicine, Faculty of Medicine Siriraj Hospital, Mahidol University, Bangkok, Thailand

## Abstract

**Background:**

COVID-19 is a pandemic disease worldwide. Although cutaneous manifestations may present in affected patients, there have been limited studies on the cutaneous findings and hair and nail abnormalities after discharge.

**Objective:**

To establish the cutaneous manifestations, hair and scalp disorders, and nail abnormalities in patients who recovered from COVID-19 infections.

**Methods:**

A retrospective chart review and telephone interviews were conducted to determine the cutaneous manifestations, hair and scalp disorders, and nail abnormalities of patients aged over 18 years who were diagnosed with COVID-19 infections at Siriraj Hospital, Bangkok, Thailand, between January and June 2020.

**Results:**

Ninety-three patients with prior COVID-19 infections participated in the study. The COVID-19 severity had been mild for most (71%). Cutaneous manifestations were reported in 8 patients (8.6%), with the common skin conditions being maculopapular rash and urticaria. The onsets of the skin conditions were before admission (1%), during admission (4.3%), and after discharge (3.2%). Increased hair shedding was also reported in 22 patients (23.7%), with a female predominance. Three patients were affected during admission, while the others were affected after discharge. The patients with moderate, severe, and critical COVID-19 infections experienced significantly more hair shedding than those with asymptomatic and mild diseases. Only 2 patients with mild COVID-19 disease reported nail abnormalities (chromonychia and brittle nails).

**Conclusions:**

Cutaneous manifestations, hair disorders, and nail abnormalities can occur in patients with COVID-19 after their discharge from hospital. Patients should therefore be followed up in anticipation of dermatological problems.

## 1. Introduction

Coronavirus disease 2019 (COVID-19), a pandemic disease that emerged in December 2019, is caused by severe acute respiratory syndrome coronavirus 2 (SARS-CoV-2) [1]. The main clinical presentation is flu-like symptoms, such as fever, cough, and sore throat. The infection can transmit into the lower respiratory tract, leading to pneumonia in some patients. In addition, organ dysfunction, acute respiratory distress syndrome, and coagulation disorders may develop, resulting in a high mortality rate. According to the COVID-19 Treatment Guidelines of the National Institutes of Health, disease severity is classified into 5 groups: asymptomatic, mild, moderate, severe, and critical [2].

Besides the respiratory system, one of the many other organs that can be affected is the skin. The cutaneous manifestations of COVID-19 can present in various clinical patterns, and their classification into the following 5 subtypes has been proposed: maculopapular rash, urticarial lesion, pseudo-chilblain, vesicular eruption and livedo, and necrosis [3]. Their onset typically occurs before and during admission, but cutaneous findings after discharge have been reported by many studies [4–10]. Although hair and nail disorders may also occur late after recovery from a COVID-19 infection, studies on the hair and nail problems of patients infected with the SARS-CoV-2 virus are rare [11–16]. Telephone interview is one tool to assess the patient's condition in this on-going COVID-19 pandemic to comply with infectious disease control policy. Thus, this study is aimed at evaluating the cutaneous manifestations, hair and scalp disorders, and nail abnormalities of patients recovered from COVID-19 infections.

## 2. Methods

Before its commencement, the study was approved by the Siriraj Institutional Review Board. This retrospective, single-center study used a combination of chart reviews and telephone interviews to establish the cutaneous manifestations, hair and scalp disorders, and nail abnormalities of patients aged over 18 years who were diagnosed with COVID-19 infections at Siriraj Hospital, Bangkok, Thailand, between January and June 2020. The COVID-19 diagnoses of all of the patients had been confirmed by positive reverse transcription polymerase chain reaction tests. A review was performed of the demographic data, clinical manifestations, treatments, and clinical courses of the COVID-19 infections, as detailed in the patients' medical records. In addition, a structured questionnaire was used to conduct telephone interviews at the end of September 2020. The study was conducted in this period because the skin manifestations in the recovery phase may occur in 3-4 months after infection, such as telogen effluvium (TE), but not too long to forget their skin conditions.

Descriptive statistics (percentages, frequency, mean, median, standard deviation, minimum, and maximum) were calculated. Fisher's exact test and the Mann–Whitney *U* test were used for qualitative and quantitative variables, respectively. Statistical significance was set at 0.05. The statistical data were analyzed using PASW Statistics for Windows (version 18.0; SPSS Inc., Chicago, Ill., USA).

## 3. Results

In all, 115 patients diagnosed with COVID-19 infections were admitted to our hospital during the study period. The demographic data and clinical characteristics of the 93 patients who participated in this study are listed in Table 1. Their mean age was 40.8 ± 15.1 years (range, 21–83 years), and the majority were males (58.1%). One-third had underlying diseases, such as hypertension, diabetes, dyslipidemia, and allergic rhinitis. Two patients had previously been diagnosed as having psoriasis, but the diseases were well controlled prior to their COVID-19 infections. The most common presentation of COVID-19 infection was upper respiratory tract symptoms. The disease severities of COVID-19 were chiefly graded as mild (71%), followed by severe (14%) and moderate (9.7%). Four patients developed acute respiratory distress syndrome, and only one patient was asymptomatic. All patients with any severity of COVID-19 infection were admitted to the hospital to monitor their clinical symptoms, to administer medications, and to prevent the spread of the disease. The median hospitalization duration was 6 days (range, 2–53 days). The median duration between the date of the COVID-19 diagnoses and the study interview was 170 days (range, 142–184 days).

### 3.1. Cutaneous Manifestations (Table 2)

Cutaneous manifestations were reported in 8 patients (8.6%), with half of those having a mild severity of COVID-19 infection. Maculopapular rash was reported in 3 patients; its onset was during admission in the case of 2 patients and 7 days after hospital discharge for the third patient. Acute urticaria was reported in 2 patients. The condition developed 3 days before the COVID-19 diagnosis in one patient with a mild disease severity, but 120 days after discharge for the other patient, who had had a critical disease state. Both patients had spontaneous remission within one week. No other potential causes of maculopapular rash or urticaria—such as a drug or an autoimmune disease—were detected in the 5 patients. Other possibly relevant cutaneous manifestations reported were petechiae and eczema. Each was found in only 1 patient with mild COVID-19 disease during admission. Psoriasis as an underlying disease was reported in 2 patients; disease aggravation occurred in one of those patients 10 days after COVID-19 discharge.

### 3.2. Hair and Scalp Disorders (Table 3)

Increased hair shedding was reported in 22 patients (23.7%). Of those, 12 patients (12.9%) reported a history of hair shedding of more than 100 hairs on regular days or more than 200 hairs on washing days. Hair shedding occurred after hospital discharge in 19 patients (87% of the affected cases), for whom the median time from the date of COVID-19 diagnosis was 42 days (range, 6–105 days). Three patients (13%) reported active hair shedding during admission, with a median time from the date of COVID-19 diagnosis of 12 days (range, 3–16 days). No other causes of anagen or telogen effluvium, such as a drug or an autoimmune disease, were detected in the 22 patients.

Increased hair shedding was found to be related with the severity of the COVID-19 disease. Patients with moderate, severe, and critical diseases had significantly greater hair shedding than those with asymptomatic and mild diseases (42.3% vs. 16.4%; *p* = 0.008; COR, 3.7; 95% CI, 1.4–10.3). Females also experienced significantly more hair shedding than males (35.9% vs. 14.8%; *p* = 0.018; COR, 3.2; 95% CI, 1.2–8.7). No other investigated results were associated with increased hair shedding.

Scalp disorders were reported in 3 patients; 2 had an aggravation of seborrheic dermatitis, and the third had deterioration of scalp psoriasis.

### 3.3. Nail Abnormalities

Only 2 patients with mild disease reported nail abnormalities. One patient observed transverse yellow-brown banding on the distal nail plates of all toenails 3 months after discharge. She denied a history of the use of concomitant medications or nail polish. The second patient complained of having brittle fingernails 3 months after discharge. Both patients still had their abnormal nails at the time of interview (about 5 months after their COVID-19 diagnoses).

## 4. Discussion

COVID-19 infection has been spreading globally as a new pandemic disease since November 2019. As our hospital is one of the largest tertiary centers in Thailand, all COVID-19 patients hospitalized there have different severities of disease. Thus, cutaneous manifestations were carefully observed by experienced physicians during admission.

An ever-increasing number of studies are reporting skin manifestations associated with COVID-19 infections. In our work, skin and hair manifestations were found in only 7 (7.5%) patients during admission, but 24 (25.8%) other cases developed after hospital discharge (19, 3, and 2 patients with hair, skin, and nail conditions, respectively). Increased hair shedding was found 1–2 months after COVID-19 infection. Maculopapular rash occurred during, or shortly after, a COVID-19 infection; this was in marked contrast with urticaria, which developed up to 5 months after infection. To date, nail changes have been reported rarely. Based on our literature review, the present study is the first to report color discoloration of the nails in a patient who had had a COVID-19 infection.

There were no cases with pseudo-chilblain or livedoid lesions, as reported in other regions, especially Europe [17]. Only urticaria and maculopapular rash were found in this study, which corresponds with the findings of other studies in tropical countries [18, 19]. This might be explained by climate differences and/or a lower severity of COVID-19 infections. Furthermore, vesicular eruption or other reported cutaneous manifestations such as erythema multiforme were not found in our study. This might be because of a small number of patients in this study.

The cutaneous lesions could be seen before admission, during admission, or after discharge. Our study found one patient with an urticarial lesion before the COVID-19 diagnosis. The lesion might have been a reaction to viral infection or a preceding symptom of COVID-19, similar to other reports [20, 21]. As to the cases whose skin rash occurred during admission, drug-induced rash should be considered a differential diagnosis from viral-related cutaneous findings. However, as no potential causative drug was found in our patients, the maculopapular rash might have been caused by the COVID-19 infection. There have been reports of late-onset skin manifestations in patients with previous COVID-19 infections, such as urticaria [4], urticarial vasculitis [5], maculopapular rash [6, 7], acute generalized exanthematous pustulosis-like [8], Kawasaki-like [9], and acral necrosis [10]. This study also found one patient developed urticaria 120 days after discharge. The association between this symptom and COVID-19 infection was unknown because of the delayed onset. A previous study reported some cutaneous manifestations associated with the severity and outcomes of COVID-19. Maculopapular rash was frequently reported in moderate severity with good outcome. Retiform purpura was associated with more severe disease and worse prognosis [22]. However, from our study, urticaria and maculopapular rash could be observed in any severity of COVID-19 except asymptomatic and had 100% survival rate.

We also studied hair disorders in patients with COVID-19 infections. Onset of increased hair shedding during admission was detected in 3 patients (3.2%), who had different severities of COVID-19 infection, ranging from mild to severe. In general, the cause of a sudden onset of hair loss might be anagen effluvium [23]. Currently, only one reported case had anagen effluvium associated with a COVID-19 infection [24]. However, this condition frequently affects almost all of the scalp hair, unlike our patients. Additionally, from our literature review, there have been no reports of hair shedding being induced by the medications used for COVID-19 therapy within a few weeks of their initiation.

In our study, the late onset of increased hair shedding was found in 20.4% of COVID-19 patients. TE would be of most concern following a systemic infection [25]. Long-term use of lopinavir/ritonavir has been reported to induce TE in human immunodeficiency virus-infected patient [26]. However, all treatments used with our COVID-19 patients were prescribed for short durations (5–14 days). Thus, TE in our patients might have resulted from viral infections or stressful events rather than drug-induced hair loss, as mentioned in other studies [12, 15, 16]. It has been proposed elsewhere that an infection or proinflammatory cytokines might induce an immediate shift from the anagen phase to the telogen phase of hair follicle growth, resulting in TE [15].

Our study revealed that two statistically significant factors determined excessive hair loss in COVID-19 patients. Firstly, the severity of the COVID-19 disease strongly influenced the risk of increased hair shedding. The greater the COVID-19 severity was, the more pronounced was the hair loss. The second factor was the gender of the patient, given that hair shedding strongly predominated in females. Thus, patients with COVID-19 infections should be warned about the possibility of hair loss and have their hair condition closely monitored during admission and after hospital discharge. This is especially important for patients who are female or have more severe infections.

Nail abnormalities associated with COVID-19 have been reported in few studies. Beau's line [11], Mee's line [13], and red half-moon nails [14] have been observed. It has been hypothesized that COVID-19 infections induce nail matrix and vascular injuries. This study is the first to report a yellow-brown chromonychia in a patient who had a mildly severe COVID-19 infection. It appeared 5 months after the onset of the COVID-19 symptoms. Chromonychia has been associated with some systemic conditions (such as Kawasaki disease), but none were found in our patient. The mechanism of chromonychia remains unclear; perhaps nail plate keratinization may be abnormal, or the chromonychia is a consequence of a vascular disorder such as vasculitis. The chromonychia may also be a late manifestation of a red half-moon nail, which has previously been reported in patient with COVID-19 infections [14]. The different colors—from red to orange to brown—may reflect a sequence of vascular inflammation followed by residual coloration after resolution. Red half-moon nails may be underrecognized in their early phase, but later found as a yellow chromonychia at the distal end of the nails. Thus, chromonychia might be one late-onset nail abnormality related with COVID-19 infections.

The limitations of this study were that it was conducted at a single center, it had a relatively small sample size, and most of its data were collected by telephone interviews. As the physicians could not assess the actual skin conditions after hospital discharge, the findings might have been influenced by recall bias. We suggest that photography and telemedicine could be used to decrease the bias gap. Furthermore, this study was conducted with an observation period about 6 months. The long-term follow-up should be considered to observe skin condition.

In conclusion, cutaneous manifestations, hair disorders, and nail abnormalities can occur after hospital discharge. Subsequent monitoring of patients is therefore advisable. Telephone interviews are one of the possible, useful, follow-up methods.

## Figures and Tables

**Table 1:** Clinical characteristics of COVID-19 infected patients.

Clinical characteristics (total = 93)	Number/total (%)
Gender	
Female	39/93 (41.9)
Male	54/93 (58.1)
Age at onset: mean ± SD (years)	40.8 ± 15.1
Underlying diseases^∗^	35/93 (37.6)
Hypertension	13/35 (37.1)
Diabetes	12/35 (34.3)
Dyslipidemia	6/35 (17.1)
Allergic rhinitis	3/35 (8.6)
Old pulmonary tuberculosis	2/35 (5.7)
Cancer	2/35 (5.7)
Stroke	2/35 (5.7)
Chronic hepatitis B infection	2/35 (5.7)
Psoriasis	2/35 (5.7)
Thalassemia	1/35 (2.9)
Alzheimer's disease	1/35 (2.9)
Presented symptoms	
Asymptomatic	1/93 (1.0)
Upper respiratory tract	66/93 (71.0)
Lower respiratory tract	26/93 (28.0)
Disease severity^ǂ^	
Asymptomatic	1/93 (1.0)
Mild	66/93 (71.0)
Moderate	9/93 (9.7)
Severe	13/93 (14.0)
Critical state	4/93 (4.3)
Laboratory investigation	
Leukopenia	25/93 (26.9)
Leukocytosis	5/93 (5.4)
Abnormal liver function test	17/91 (18.7)
Elevated C-reactive protein	35/80 (43.8)
Abnormal chest X-ray	26/93 (28.0)
Duration of hospitalization: median (min, max; days)	6.0 (2.0, 53.0)

^∗^One patient could have more than one disease. ^ǂ^The severity of COVID-19 was classified according to the COVID-19 Treatment Guidelines of the National Institutes of Health.

**Table 2:** Cutaneous manifestations in COVID-19 patients.

Cutaneous manifestations	Onset of rashes	Number of patients	Duration from date of COVID-19 diagnosis (days)	Severity of COVID-19	Medications during onset of skin manifestation	Duration of rashes	Treatment
Relevant manifestations							
Urticaria	Before admission	1	3	Mild	No	1 day	No
	After discharge	1	150 (after discharge 120 days)	Critical	No	7 days	No

Maculopapular rash	During admission	1	2	Moderate	(i) Lpv/r 400/200 mg/d (day 3) (ii) CQ 500 mg/d (day 3)	2 weeks	(i) Desoximetasone cream (ii) Cetirizine (iii) Loratadine
		1	10	Severe	(i) Lpv/r 400/200 mg/d (5 days) (ii) CQ 500 mg/d (5 days) (iii) DRV 1,200 mg/d (day 5) (iv) RTV 200 mg/d (day 5) (v) HCQ 1,000 mg/d (day 5) (vi) FVR 1,200 mg/d (day 7)	5 days	(i) 0.1% triamcinolone cream
	After discharge	1	12 (after discharge 7 days)	Mild	No	3 days	No

Possibly relevant manifestations							
Petechiae	During admission	1	2	Mild	No	2 days	No
Eczema	During admission	1	2	Mild	No	2 days	No

Other manifestations							
Aggravation of psoriasis (plaque type)	After discharge	1	23 (after discharge 10 days)	Severe	No	2 weeks	(i) 10%urea + 0.1% triamcinolone cream (ii) 10%urea + betamethasone (iii) 0.02% triamcinolone cream (iv) Tar shampoo

CQ: chloroquine; DRV: darunavir; FVR: favipiravir; HCQ: hydroxychloroquine; Lpv/r: lopinavir/ritonavir; RTV: ritonavir.

**Table 3:** Hair disorders in COVID-19 patients.

Clinical manifestations and laboratory investigations: Number/total (%)	Increased hair shedding	No increased hair shedding	Crude OR (95% CI)^∗^	Adjusted OR (95% CI)	*p* value
Severity of COVID-19 (*n* = 93)	22 (23.7)	71 (76.3)			
Asymptomatic-mild (*n* = 67)	11 (16.4)	56 (83.6)	3.7 (1.4, 10.3)	5.8 (1.8, 18.9)	0.008
Moderate-severe-critical state (*n* = 26)	11 (42.3)	15 (57.7)			
Gender					
Female (*n* = 39)	14 (35.9)	25 (64.1)	3.2 (1.2, 8.7)	5.0 (1.6, 15.9)	0.018
Male (*n* = 54)	8 (14.8)	46 (85.2)			
Age at onset: mean ± SD (years)	43.5 ± 16.8	40.0 ± 14.5			0.339
Laboratory investigations					
Leukopenia (*n* = 25)	3 (12.0)	22 (88.0)			0.222
Leukocytosis (*n* = 5)	2 (40.0)	3 (60.0)			
Abnormal liver function test (*n* = 17)	6 (35.3)	11 (64.7)			0.209
Elevated C-reactive protein (*n* = 35)	12 (34.3)	23 (65.7)			0.091

^∗^The data are expressed as odds ratio with 95% confidence interval. ^∗∗^Multivariable odds ratio was calculated by stepwise logistic regression. OR: odds ratio; CI: confidence interval.

## Data Availability

The data used to support the findings of this study are available from the corresponding author upon request.
